# Beyond Archaea: The Table Salt Bacteriome

**DOI:** 10.3389/fmicb.2021.714110

**Published:** 2021-10-29

**Authors:** Leila Satari, Alba Guillén, Adriel Latorre-Pérez, Manuel Porcar

**Affiliations:** ^1^Institute for Integrative Systems Biology (I2SysBio), Universitat de València-CSIC, Paterna, Spain; ^2^Darwin Bioprospecting Excellence S.L., Parc Científic Universitat de València, Paterna, Spain

**Keywords:** table salt microbiome, halotolerant bacteria, halophilic bacteria, haloarchaea, 16S rRNA gene sequencing analysis

## Abstract

Commercial table salt is a condiment with food preservative properties by decreasing water activity and increasing osmotic pressure. Salt is also a source of halophilic bacteria and archaea. In the present research, the diversity of halotolerant and halophilic microorganisms was studied in six commercial table salts by culture-dependent and culture-independent techniques. Three table salts were obtained from marine origins: Atlantic Ocean, Mediterranean (Ibiza Island), and Odiel marshes (supermarket marine salt). Other salts supplemented with mineral and nutritional ingredients were also used: Himalayan pink, Hawaiian black, and one with dried vegetables known as Viking salt. The results of 16S rRNA gene sequencing reveal that the salts from marine origins display a similar archaeal taxonomy, but with significant variations among genera. Archaeal taxa *Halorubrum*, *Halobacterium*, *Hallobellus*, *Natronomonas*, *Haloplanus*, *Halonotius*, *Halomarina*, and *Haloarcula* were prevalent in those three marine salts. Furthermore, the most abundant archaeal genera present in all salts were *Natronomonas*, *Halolamina*, *Halonotius*, *Halapricum*, *Halobacterium*, *Haloarcula*, and uncultured *Halobacterales*. *Sulfitobacter* sp. was the most frequent bacteria, represented almost in all salts. Other genera such as *Bacillus*, *Enterococcus*, and *Flavobacterium* were the most frequent taxa in the Viking, Himalayan pink, and black salts, respectively. Interestingly, the genus *Salinibacter* was detected only in marine-originated salts. A collection of 76 halotolerant and halophilic bacterial and haloarchaeal species was set by culturing on different media with a broad range of salinity and nutrient composition. Comparing the results of 16S rRNA gene metataxonomic and culturomics revealed that culturable bacteria *Acinetobacter*, *Aquibacillus*, *Bacillus*, *Brevundimonas*, *Fictibacillus*, *Gracilibacillus*, *Halobacillus*, *Micrococcus*, *Oceanobacillus*, *Salibacterium*, *Salinibacter*, *Terribacillus*, *Thalassobacillus*, and also Archaea *Haloarcula*, *Halobacterium*, and *Halorubrum* were identified at least in one sample by both methods. Our results show that salts from marine origins are dominated by Archaea, whereas salts from other sources or salt supplemented with ingredients are dominated by bacteria.

## Introduction

Sodium chloride, table salt, is widely used in the food industry as a taste, texture, and flavor enhancer (Henney et al., 2010a), stabilizer, and food preservative (Albarracín et al., 2011). Most processed products contain sodium chloride as a preservative, which strongly affects osmotic pressure (Albarracín et al., 2011). In the process of salting, sodium and chloride can interact with water molecules and reduce the water activity of the food (Tim, 2002). Therefore, salinity can prevent microbial spoilage (Henney et al., 2010b). Additionally, salinity decreases oxygen solubility, thus controlling aerobic growth. Moreover, high amounts of sodium and chloride can interfere with the enzymatic activity of some microorganisms (Man, 2007). Finally, in the presence of salt, microbial cells spend more energy by pumping sodium out from their cells to cope with the harsh effect of the osmotic shock (Henney et al., 2010b).

Saline environments, such as solar ponds, saline lakes, brine springs, rock salts, and seawater, are sources of commercial table salt (Antonites, 2020). Most commercial salts are refined and finely ground before distribution. Saline environments can also be the origin of salt-loving microorganisms, halophiles (Oren, 2015). Therefore, those microbes might be entrapped in the fluid inclusions during the crystallization process and remain viable after extraction and packaging (Baati et al., 2010).

In saline habitats, archaea and halophilic bacteria are the predominant microbial communities. Those extremophiles are classified into several subgroups according to their optimum salt requirements (DasSarma and DasSarma, 2017). Most of the halophilic microorganisms belong to the slight (0.2–0.85 M) and moderate (0.85–3.4 M) halophiles. However, some extreme halophiles have been detected in hypersaline environments where the salt concentration lies between 3.4 and 5.1 M (DasSarma and DasSarma, 2017). Moreover, some studies described another subgroup for halophiles; those can thrive under the borderline range of salinity (1.5–4.0 M salt) (Ghosh et al., 2019). Some halotolerant bacteria can also tolerate low salinity and even non-saline environments. Halophiles have developed different adaptations to cope with the saline environment (Harding et al., 2016). In the “salt in” strategy (Koller, 2019), the presence of K^+^ and, less frequently, Na^+^ is central to balance the intracellular osmotic pressure with that of the environment (Roberts, 2005). Additionally, some halophiles balance their intracellular osmotic pressure by accumulating organic compatible solutes in the cytoplasm (Roberts, 2005). Besides this, most of the extreme halophiles belong to the domain of Archaea, whose optimum salt requirements are usually more than 25% w/v dissolved salts (Bowers and Wiegel, 2011). Among those, Haloarchaea have a higher content of acidic amino acids and a lesser amount of hydrophobic amino acids in their enzymes and proteins, respectively (Oren, 2002; Leigh et al., 2011).

Table salt or very salty food are sources of halophiles, especially halophilic bacteria, a part of which may originate from the original location from which salt was extracted. Considering this, processed food can be seen as a habitat for halophiles provided that the salt levels are high (Lee, 2013). Indeed halophiles are found in fermented seafood (Das et al., 2020), cheeses (Kothe et al., 2020), sauces (Sagdic et al., 2017; Ohshima et al., 2019), green table olives (Randazzo et al., 2017), and pickles (Stoll et al., 2020). Some halophilic and halotolerant microorganisms can be established in the human gut microbiome, and the gut halophilic microbiota is considered to be linked to some chronic diseases (Seck et al., 2019). In patients with obesity, type 2 diabetes, celiac disease, or inflammatory bowel disease, an increased frequency of these microorganisms has been detected in samples from the intestine, colon, and stool compared to healthy individuals (Seck et al., 2019; Biswas and Rahaman, 2020).

There are very few reports on the microbiome of table salt, and they tend to focus on the archaeal taxa (Henriet et al., 2014; Gibtan et al., 2017). The present work describes a complete characterization of the bacteriome of six table salts by using culture-dependent and culture-independent techniques. We have studied the microbial contents of different commercial table salts, from regular ones to some with added spices or minerals and from salts of marine origin to some originating from salt mines, and identified the distribution of both archaea and bacteria isolates and sequences among the studied salts.

## Materials and Methods

### Table Salt Samples

**FIGURE 1:**
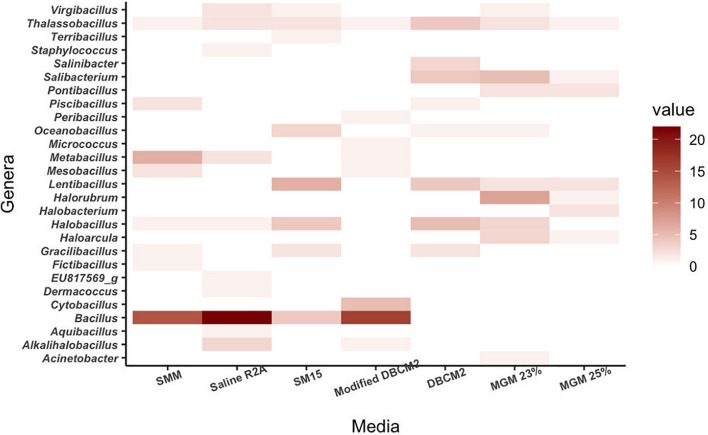
Bacterial and archaeal genera from table salts recovered on culture media with different salt concentrations and nutritional compositions. SMM and saline R2A are supplemented with 10% w/v total salt, while their nutritional compositions are different. SM15 contains 17% w/v total salt. Modified DBCM2 and DBCM2 have 2.5% w/v total salt, 25% w/v total salt, respectively. MGM was also especially used for the isolation of archaea, supplemented with 23 and 25% w/v total salt. The color code indicates the number of microorganisms isolated on each medium.

Seven commercial table salts were analyzed in this study. The salts were purchased from local supermarkets (el Corte Inglés and Mercadona, Valencia, Spain) and stored at room temperature in a dark and dry place. These salts can be grouped in salts from marine origin (Atlantic salt, Ibiza salt, and supermarket marine salt), larger granulated salts supplemented with other ingredients (two black salts and Viking salt), and salt from inland salt mines (Himalayan pink salt). The visual characteristics of food-grade salts such as color, size of their particles, origins, and other features are shown in Supplementary Table 1 and Figure 1. In order to have a homogenized microbial diversity all over the packages, the salt particles were thoroughly mixed before sampling.

### Solutions and Media

Media and solutions were used to isolate different halotolerant and halophilic microorganisms based on their salt requirements. SMM and saline R2A are supplemented with approximately 100 g^–*l*^ total salt, while their nutritional compositions are different. SM15 contains almost 170 g^–*l*^ total salt. Modified DBCM2 and DBCM2 have 25 and 250 g^–*l*^ total salt, respectively.

• Sea salt solution (g^–*l*^): NaC1, 81; MgC1_2_, 7.0; MgSO_4_, 9.6; CaCl_2_, 0.36; KCI, 2.0; NaHCO_3_, 0.06; NaBr, 0.026. The sea salt solution contains almost 10% (w/v) total salts (Rodriguez-Valera et al., 1981).
• Sea water (SW) solution (g^–*l*^): NaC1, 240; MgC1_2_⋅6H_2_O, 30.0; MgSO_4_, 35.0; KCI, 7.0; NaHCO_3_, 0.2; NaBr, 0.8, 1 M Tris⋅Cl, pH 7.5. The SW solution contains approximately 30% (w/v) total salts (Dyall-Smith, 2009).
• SMM medium (g^–*l*^): NaCl, 46.8; MgCl_2_⋅6H_2_O, 19.5; MgSO_4_⋅7H_2_O, 30.5; CaCl_2_, 0.5; KCl, 3.0; NaHCO_3_, 0.1; NaBr, 0.35; casein digest, 5.0; sodium pyruvate, 1.1; and agar, 15.
• Saline R2A medium (g^–*l*^): peptone, 1; yeast extract, 0.5; dextrose, 0.5; soluble starch, 0.5; K_2_HPO_4_, 0.3; MgSO_4_, 0.05; and sodium pyruvate; 0.3, which is supplemented with 10% sea salt solution (Rodriguez-Valera et al., 1981).
• SM15 medium (g^–*l*^): NaCl, 117; MgCl_2_⋅6H_2_O, 19.5; MgSO_4_⋅7H_2_O, 30.5; CaCl_2_, 0.5; KCl, 3.0; NaHCO_3_, 0.1; NaBr, 0.35; casein digest, 5.0; yeast extract, 2.5; sodium pyruvate, 1.1; glucose, 1.0; and agar, 18 (León et al., 2014).
• DBCM2 medium: DBCM2 medium was prepared by mixing 833 ml 30% SW solution with 167 ml low nutritional solution with the following compositions: sodium pyruvate, 0.11 g; glucose, 0.0025 g; peptone, 0.0125 g; yeast extract, 0.0125 g; and agar, 20.0 g (Henriet et al., 2014).
• Modified DBCM2 medium: This medium was prepared by mixing 833 ml 0.1 × SW solution with 167 ml of the same low nutritional solution and agar. This medium is optimized for the growth of halotolerant bacteria.

The media were selected based on the similarity of their minerals to the seawater, and the pH of all was adjusted to 7.5 ± 0.05 with 1 M KOH or 1 M HCl.

Moreover, two following modified growth media (MGMs) were specially used to isolate haloarchaea. Modified growth media supplemented with 23 and 25% SW solutions were prepared by mixing 767 and 833 ml 30% SW solution with 200 and 134 ml pure water, respectively. Each medium is supplemented with the following (g^–*l*^): peptone (oxide), 5.0; yeast extract, 1.0; and agar, 15.0. The pH of the MGMs was adjusted to 7.5 with 1 M Tris⋅Cl, pH 7.5, and its final volume was adjusted to 1,000 ml. The only difference between these two media is the total salt concentrations. MGM with 23% salt is an appropriate medium to recover various halophilic bacteria However, the genus *Halobacterium* can be retrieved on 25% MGM (Dyall-Smith, 2009).

### Culture-Dependent Approach

For culture-based analysis, 10 g of each salt was added to 40 ml sterile Milli-Q H_2_O in a 50-ml Falcon tube (final concentration of the saline solution: 25%, w/v). Salt was dissolved by vortexing, and then the saline solution (except the saline solution from Viking salt) was centrifuged for 45 min at 6,000 rpm and 4°C, and the pellets were resuspended in 2 ml phosphate-buffered saline (PBS) buffer (1×, pH 7.4). Serial dilutions were prepared by resuspending 10 μl of the bacterial suspension in the PBS buffer (diluted 1:10) and plated on SMM, saline R2A, SM15, DBCM2, modified DBCM2, and MGMs. The plates were sealed with parafilm and incubated at 30°C (for 23 and 25% MGMs, at 37°C) for at least 10 weeks. Other saline solutions, such as PBS buffer and sea salt solution [10% (w/v) total salts], were also used for the isolation of bacteria and archaea from table salts.

In the case of saline solution from Viking salt, it was centrifuged at 500 rpm for 10 min at 4°C, and its bulky insoluble particles were removed. Then, the supernatant was transferred to another 50-ml Falcon tube, and the extraction was continued as described above.

Individual colonies were selected based on the pigmentation and morphology of the colonies, and they were re-streaked on fresh media. Finally, glycerol stocks were prepared from the fresh biomass from the plates in 20% glycerol (v/v) supplemented with a saline medium and stored at –80°C.

Universal 16S rRNA gene primers 8F (5′-AGAGTTTGATC CTGGCTCAG-3′), 1492R (5′-CGGTTACCTTGTTACGACTT-3′) for bacteria, and A616D (5′-CGKTTGATCCTGCCGGA-3′) and P1525R (5′-WAGGAGGTRATCCADCC-3′) for Archaea were used for PCR. Qiagen master mix 2 × was used for all reactions. A loopful of a fresh culture growing on solid medium was resuspended in 100 μl of Milli-Q H_2_O. The suspension was pre-incubated at 100°C for 10 min, and 1 μl of the suspension was used as the DNA template. PCR for bacteria and archaea was performed based on the following program: initial step of incubation at 94°C for 3 min followed by 30 cycles of denaturation at 94°C (30 s), annealing at 50°C (30 s), and an extension step at 72°C (1 min 30 s). The last stage was the final extension step at 72°C for 10 min. The amplification of the targeted gene fragment was monitored by 1% agarose gel electrophoresis. Then, dsDNA was purified from the PCR products and resuspended in 10 μl MilliQ H_2_O. Sanger sequencing was performed by tagging with BigDye Terminator v3.1 Cycle Sequencing Kit (Applied Biosystems, Carlsbad, CA, United States) at the Sequencing Service (SCSIE) of the University of Valencia. All sequences were edited and compared with the EzBioCloud online database^1^ for bacteria and Archaea.

### Culture-Independent Approach

DNeasy PowerSoil Pro Kit (Qiagen, Germany) was used for DNA extraction from food-grade salt samples. In the preparation steps, 20 g of each sample was completely dissolved in 100 ml sterile Milli-Q H_2_O (20% w/v) and centrifuged for 45 min at 6,000 rpm and 4°C to collect microbial cells. The pellets were resuspended in 100-μl solution C1 and transferred into 2-ml bead tubes, and extraction was carried out according to the manufacturer’s instructions. As a negative control, 100 ml sterile Milli-Q H_2_O (salt-free) was centrifuged, and DNA extraction was carried out in the same way as the DNA extraction for the saline samples. The quantity of the extracted DNA was analyzed through the Qubit dsDNA HS Assay kit (Qubit 2.0 Fluorometer, Q32866).

For 16S rRNA gene sequencing analysis, the V3–V4 and V4–V5 regions were targeted for bacteria and archaea, respectively. Primers 341F (5′-CCTAYGGGRBGCASCAG-3′) and 806R (5′-GGACTACNNGGGTATCTAAT-3′) were used to amplify the bacterial 16S rRNA gene, while primers Arch519F (5′-CAGCCGCCGTAA-3′) and Arch915 (5′-CGTGCTCCCCCGCCAATTCCT-3′) were used for the archaeal 16S rRNA gene (Klindworth et al., 2013). All PCR reactions were carried out with Phusion High-Fidelity PCR Master Mix (New England Biolabs). The PCR products were mixed at equal density ratios. The pool was then purified with the Qiagen Gel Extraction Kit (Qiagen, Germany). Sequencing libraries were generated with NEBNext Ultra^TM^ DNA Library Prep Kit for Illumina and quantified *via* Qubit and q-PCR. Finally, the NovoSeq 6000 Sequencing System (2 × 300 bp) was employed for sequencing the samples at Novogene (Cambridge, United Kingdom). Raw Illumina reads were analyzed *via* Qiime2 software (v. 2020.8) (Bolyen et al., 2019). The Demux plugin was used to assess the quality of the reads, and the Qiime2-integrated DADA2 pipeline was employed for trimming and joining the sequences, removing chimeras, and detecting amplicon sequence variants (ASVs; > 99.9% similarity). The classify-Sklearn module (feature-classifier plugin) was applied to assess the taxonomy of each sequence variant, using the SILVA (v. 138) database as a reference.

Data were subsequently analyzed by using different R packages. Rarefaction curves were constructed with the iNEXT and ggiNEXT functions (iNEXT) (Hsieh et al., 2016). Principal coordinates analysis (PCoA) was created with the plot_ordination function (phyloseq) (McMurdie and Holmes, 2013) using Bray–Curtis dissimilarities as a distance method. Taxonomic barplots were created with the following R libraries: ggplot2, forcats, and tidyr.

## Results

### Culture-Dependent Approach

A strain collection was set by culturing table salt samples on a range of saline media, with a broad range of salinity and different nutritional and trace elements, and after incubation at 30 and 37°C for more than 2 months (see “Materials and Methods” section). This allowed us to isolate 79 different strains from 28 bacterial and archaeal taxa (Supplementary Table 2). Comparing the culturomics results with the previous studies, of those, 27 isolates belonged to halotolerant and slightly halophilic bacteria, 45 strains belonged to moderately halophilic bacteria, and one genus corresponded to extremely halophilic bacteria, whereas six archaeal strains were isolated and identified. The bacteria were mostly isolated during the first 4 weeks of incubation, while the archaeal colonies were, in general, more frequent within the small colonies that were only noticeable after 5 weeks of incubation. Interestingly, all strains were isolated from table salts by dissolving salt samples in sterile Milli-Q H_2_O. Using other saline solutions was not efficient enough to recover this vast microbial biodiversity. However, most bacteria were isolated by this method; especially slightly and moderately halophilic bacteria were spore-forming strains. Surprisingly, no colonies from the Himalayan black salt were observed in any media, while only three genera could be isolated from the Hawaiian black salt on saline R2A media with 10% (w/v) total salt concentration. Most of the bacterial isolates from Viking salt were isolated on the media with less than 17% (w/v) total salt. Regarding bacterial isolates from media with very high salt concentrations, *Acinetobacter* sp. was the only genus isolated on the MGM with 23% (w/v) salt. From Ibiza salt, a few extremely halophilic bacteria were isolated on the DBCM2 medium. The genus *Bacillus* was the most frequent taxon isolated on four of the media.

The microbial diversity of the commercial table salts, by using various media, is shown in Figure 1. Surprisingly, no *Bacillus* sp. was identified among the colonies growing on the media with high salt concentrations (DBCM2 and MGMs), while *Salibacterium* sp. and *Pontibacillus* sp. were mostly isolated on media with more than 20% salinity. More interestingly, *Thalassobacillus* sp. was identified in isolates from a broad range of salinity [2.5–25% (w/v) salt concentrations]. The genus *Lentibacillus* was only recovered from the moderate and hyper-saline media (SM15, DBCM2, and MGMs). By using MGM supplemented with 23% (w/v) salt, we were able to recover a variety of halophilic bacteria, such as *Virgibacillus*, *Oceanobacillus*, *Halobacillus*, and *Acinetobacter*, while none of those genera was recovered on 25% MGM. *Salinibacter* sp. was isolated only from the DBCM2 medium, while the genera *Aquibacillus* and *Terribacillus* were isolated from saline R2A and SM15 media, respectively. Some potential pathogens, *Cytobacillus* sp. and *Peribacillus* sp., were also isolated from the media with 2.5% (w/v) total salt concentration. Finally, human-associated bacteria, such as *Fictibacillus* and *Dermacoccus*, were found on SMM and saline R2A media, respectively.

**FIGURE 2:**
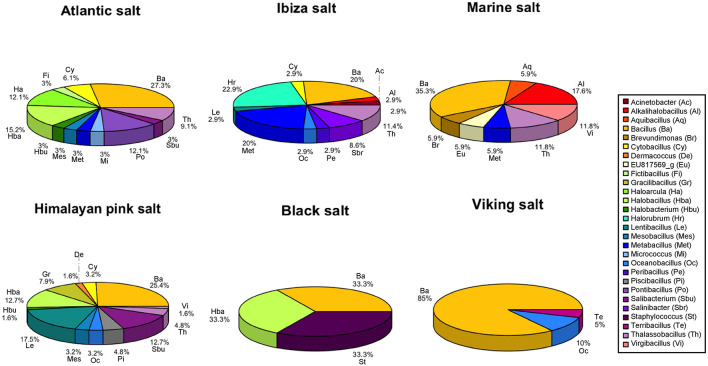
Microbial profiles of six table salt samples based on the identification of culturable isolates. The percentage indicates that the value was calculated based on the total number of isolates in each table salt. The abbreviation is correlated to the genus name: Ac, *Acinetobacter*; Al, *Alkalihalobacillus*; Aq, *Aquibacillus*; Ba, *Bacillus*; Br, *Brevundimonas*; Cy, *Cytobacillus*; De, *Dermacoccus*; Eu, EU817569_g; Fi, *Fictibacillus*; Gr, *Gracilibacillus*; Ha, *Haloarcula*; Hba, *Halobacillus*; Hbu, *Halobacterium*; Hr, *Halorubrum*; Le, *Lentibacillus*; Mes, *Mesobacillus*; Met, *Metabacillus*; Mi, *Micrococcus*; Oc, *Oceanobacillus*; Pe, *Peribacillus*; Pi, *Piscibacillus*; Po, *Pontibacillus*; Sbu, *Salibacterium*; Sbr, *Salinibacter*; St, *Staphylococcus*; Te, *Terribacillus*; Th, *Thalassobacillus*; Vi, *Virgibacillus*.

The genus *Bacillus*, with more than 50 isolates, was the most abundant genera in all table salt samples and was particularly frequent among the Viking salt isolates (Figure 2). The genera *Halobacillus* and *Lentibacillus* were also frequent genera with 14 isolates, and they were isolated from three different commercial salts. However, both genera were the most frequent taxa recovered from the Himalayan pink salt sample with eight and 12 isolates, respectively. *Thalassobacillus* sp. was another abundant genus with 13 isolated bacteria in our collection. This genus was identified from four commercial salts and was not isolated from black salt nor Viking salt. The genera *Salibacterium* and *Metabacillus* were isolated from Himalayan pink salt and Ibiza salt with nine and seven culturable isolates, respectively. *Lentibacillus* sp. and *Oceanobacillus* sp. were also recovered from Ibiza salt and Himalayan pink salt. At the genus level, *Aquibacillus* sp. and *Gracilibacillus* sp. were only isolated from supermarket salt and Himalayan pink salt, respectively, while genus *Terribacillus* was only found in the Viking salt.

The highest bacterial diversity was observed within the strains isolated from the Himalayan pink salt, followed by Atlantic and Ibiza salts. Although the microbial compositions of these salts were different, the shared microbial profile among these three samples were *Bacillus* sp., *Cytobacillus* sp., and *Thalassobacillus* sp. Moreover, some genera, *Pontibacillus* sp., *Acinetobacter* sp., and *Piscibacillus* sp., were only isolated from the Atlantic, Ibiza, and Himalayan pink salt, respectively. Interestingly, haloarchaea *Halorubrum* sp. and extremely halophilic bacteria *Salinibacter* sp. were only isolated from the Ibiza salt besides other bacteria: *Acinetobacter*, *Alkalihalobacillus*, *Bacillus*, *Cytobacillus*, *Halorubrum*, *Lentibacillus*, *Metabacillus*, *Oceanobacillus*, *Peribacillus*, and *Thalassobacillus*. Moreover, seven genera were recovered from the supermarket marine salt: *Alkalihalobacillus*, *Aquibacillus*, *Bacillus*, *Brevundimonas*, *Metabacillus*, *Thalassobacillus*, and *Virgibacillus*, whereas black salt displayed the lowest microbial diversity of culturable strains from the genera *Bacillus*, *Halobacillus*, and *Staphylococcus*.

Finally, haloarchaea were isolated from three commercial table salts. The genus *Halobacterium* was isolated from Atlantic salt and Himalayan pink salt. However, *Haloarcula* sp. and *Halorubrum* sp. were only found in Atlantic and Ibiza salts, respectively. The taxon *Halorubrum*, with eight phylotypes belonging to three species, was the most frequent archaea in the Ibiza salt.

### Culture-Independent Approach

**FIGURE 3:**
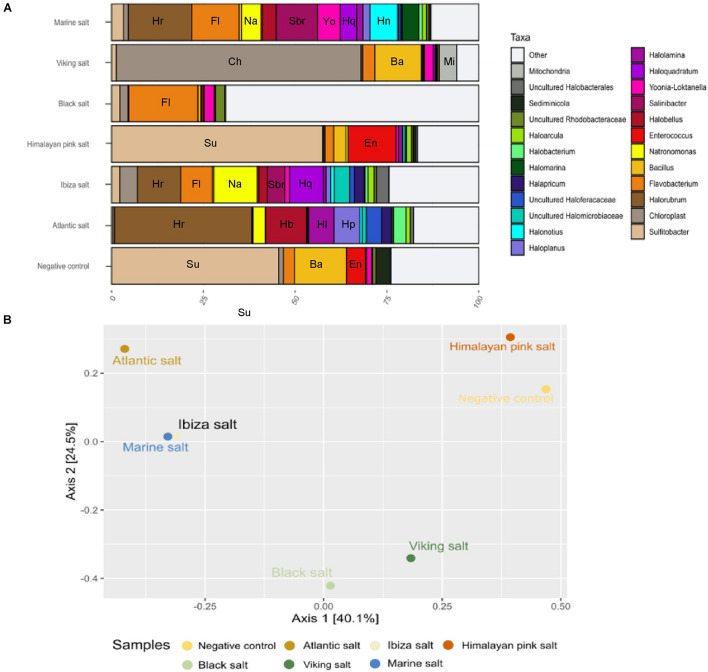
**(A)** Microbial composition of table salt samples after 16S rRNA gene sequencing. The abbreviation is correlated to the genus name and highlighted the most frequent genera: Su, *Sulfitobacter*; Ba, *Bacillus*; En, *Enterococcus*; Hr, *Halorubrum*; Hb, *Halobellus*; Hl, *Halolamina*; Hp, *Haloplanus*; Fl, *Flavobacterium*; Na, *Natronomonas*; Sbr, *Salinibacter*; Hq, *Haloquadratum*; Ch, chloroplast; Mi, mitochondria; Yo, *Yoonia_Loktanella*; Hn; *Halonotius*. **(B)** Beta diversity (PCoA) based on Bray–Curtis dissimilarity metric. Distances to the linear statistical correlation indicate the similarity of the microbial diversity of each sample affected by the origin of those salts.

Of seven selected commercial table salts, we were able to extract a sufficient amount of DNA in six of them. These six commercial salts, labeled as Atlantic salt, Ibiza salt, Himalayan pink salt, Hawaiian black salt, Viking salt, and supermarket marine salt, are shown in Supplementary Figure 1 and were analyzed through 16S rRNA gene sequencing. By using the Archaea-specific primers (see “Materials and Methods” section), both archaea and bacteria were detected in the food-grade salts. The archaeal and bacterial diversity of the six table salts showed that the Ibiza salt sample displayed the highest biodiversity, with 335 identified ASVs in total. In general, the main archaeal genera were *Halorubrum* (37.4% in Atlantic salt), *Natronomonas* (12% in Ibiza salt), *Halobellus* (11.2% in Atlantic salt), *Haloquadratum* (9.2% in Ibiza salt), *Haloplanus* (7% in Atlantic salt), and *Halonotius* (7.5% in supermarket marine salt). Other frequent archaea genera present in all samples were *Natronomonas*, *Halolamina*, *Halonotius*, *Halapricum*, *Halobacterium*, *Haloarcula*, and uncultured Halobacterales (Figure 3A). The salt samples from the Atlantic Ocean and Mediterranean Sea (Ibiza island) were mainly composed of archaea (approximately 90 and 70% of the total microbial compositions, respectively). On the other hand, the supermarket marine salt sample (a salt whose production comes from the Odiel marshes in the province of Huelva, close to the Atlantic Ocean) displayed a high presence of bacterial genera, such as *Salinibacter*, *Flavobacterium*, or *Yoonia-Loktanella* with a frequency of 11.3, 12.9, and 6.2%, respectively.

The Himalayan pink salt, black salt, and Viking salt presented a taxonomic profile dominated by bacteria. More than 85% of their taxonomic composition corresponded to genera such as *Sulfitobacter*, *Flavobacterium*, *Bacillus*, *Enterococcus*, or *Salinibacter*. However, it should be noted that the genus *Sulfitobacter*, which was found in all these three samples, was very abundant (57.5%) in the Himalayan pink salt, while in others it did not exceed 3%. Regarding the genus *Salinibacter*, we observed that it was more frequent in samples from the Mediterranean Sea than in the remaining salts. Viking salt also contained chloroplast sequences, as this salt has pepper and dry onion as ingredients.

Beta diversity analysis (PCoA) showed that the samples tend to form three different groups (Figure 3B). The first groups were composed by salts with mainly marine origins. The second group consisted of salts of non-marine origins. Finally, the Himalayan pink salt shared a similar taxonomic profile with the negative control for some genera. This clustering suggests that there is a correlation between the taxonomic profile similarities and the origin of the salt among pure marine salts.

**FIGURE 4:**
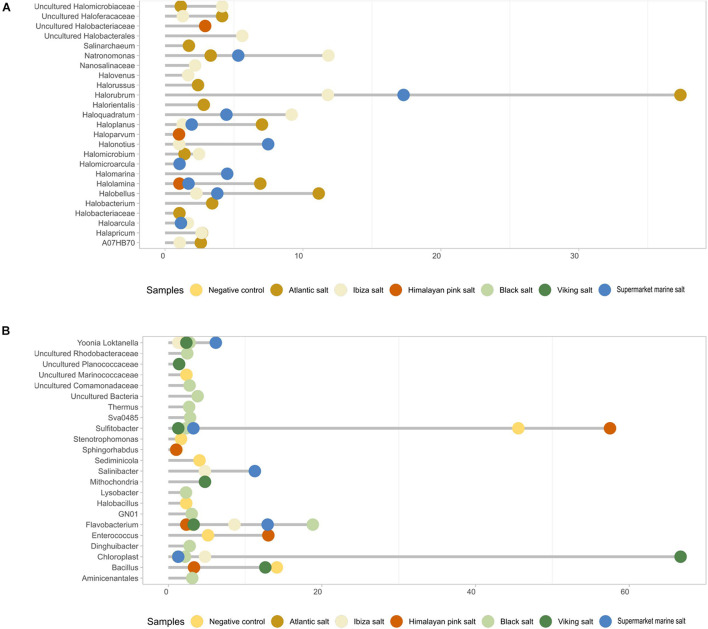
Main archaeal composition **(A)** and bacterial composition **(B)** of each table salt sample based on the 16S rRNA gene metagenomic analyses with archaea-specific primers. Only values higher than 1% are shown to explain the biodiversity of the samples.

We further investigated the distribution among the samples of archaea and bacteria with an average abundance higher than 1% (Figure 4). The variation in the taxonomic profiles was higher in the case of archaea than for bacteria. A higher number of archaea was detected in samples coming from the Atlantic Ocean, the Ibiza Island, and the Odiel marshes. The most abundant taxon in Atlantic, Ibiza, and supermarket marine salts was *Halorubrum*, followed by *Hallobellus*, *Natronomonas*, *Haloplanus*, and *Haloarcula* (Figure 4A). The genus *Natronomonas* presented a relative abundance of 11.8% in the Ibiza salt sample. Nevertheless, in the other marine salt samples, the abundances ranged between 3.3 and 5.3%. Finally, the genus *Haloarcula* was present in all three types of salt, but with values of around 1.6%.

Other relevant genera included *Halonotius* and *Halomarina*, which were especially abundant in the supermarket marine salt (7.5 and 4.5% of relative abundance). In addition, the genus *Halolamina* (7% in the Atlantic salt sample) and, to a lower extent, an uncultured *Haloferacaceae* were detected. For the Ibiza salt sample, the genus *Haloquadratum* was the most abundant archaea (9.2%), followed by *Halobacterales* (5.6%). Interestingly, the Himalayan pink salt was the unique non-marine sample in our collection where some archaea were detected. These genera were *Halolamina* (as in the Atlantic and supermarket salt sample), *Haloparvum* (1%), and some genera from the family *Halobacteriaceae* (2.9%).

The main bacterial genera for each sample were also analyzed (Figure 4B). Except for *Sulfitobacter* and *Flavobacterium*, the rest of the genera displayed less than 20% abundance. The most frequent bacterial genera were *Sulfitobacter* (present in all samples, except in the Atlantic salt), *Bacillus* (12.7% in Viking salt, but similar in negative control), *Enterococcus* (13% in Himalayan pink salt), Flavobacterium (mainly in the black salt sample), and Salinibacter (only in the marine-like salts). It should be noted that the Atlantic was the only marine salt sample where no bacterial genera was over 1% in relative abundance. The supermarket marine and Ibiza salts were rich in the following genera: *Flavobacterium* (12.9 and 8.6%), *Salinibacter* (11.3 and 4.8%), *Yoonia*-*Loktanella* (6.2 and 1.3%), and *Sulfitobacter* (3.3 and 2.3%). The genus *Sphingorhabdus* was present in the Ibiza sample and in the Himalayan pink salt, a salt whose microbiome was mainly composed of *Sulfitobacter* (57.5%) and *Enterococcus* (13%).

Since the overall DNA concentration obtained from the salts was very low, a negative control of the extraction was also included and sequenced in order to shed light on the possible contaminations in the results of the salt samples.

The negative control displayed high frequencies of the genera *Sulfitobacter* (45.6%), *Bacillus* (14.1%), *Enterococcus* (5.2%), and, to a lesser extent, *Flavobacterium*, *Yoonia*-*Loktanell*, uncultured *Marinococcaceae*, *Halobacillus*, and *Stenotrophomonas*, with relative abundances between 2.3 and 4%. Some genera were only found in some salts—for example, the genus *Planococcaceae* was only detected in Viking salt, while uncultured *Comamonadaceae*, *Dinghuibacter*, *Aminicenantales*, *Lysobacter*, *Thermus*, and the clade candidates Sva0485 (deltaproteobacteria) and GN01 were only present in black salt.

The proportion of archaea in the original sample is usually lower than the abundance of bacteria. Besides the archaea-specific primers, bacteria-specific primers were used to study both bacterial and archaeal communities. Although there is a bias by using archaea-specific primers for amplifying bacteria and *vice versa*, the results showed that the bacterial profiles obtained by using archaea- and bacteria-specific primers were comparable. Nevertheless, the relative abundance of some genera differed due to the amplification bias. The main bacterial and archaeal genera identified with the bacteria-specific primers for the Viking, Ibiza, and Atlantic salts are also shown in Supplementary Figure 2.

## Discussion

We report here a complete characterization of the bacterial and archaeal communities of six table salts by 16S rRNA gene sequencing analysis and culturomics. The next-generation sequencing analysis revealed that the three marine salts were rich in archaea, while the Himalayan pink salt, black salt, and Viking salt were rich in bacteria. It was evidenced that our collection of samples could be divided into two groups: one made up of salts from marine environments where the main presence of archaea stood out and a group made up of non-marine salts with high relative abundances of bacteria.

The total DNA extraction for 16S rRNA gene that we carried out resulted in very low amounts of DNA even after several steps of enrichment. Thus, we extracted the DNA from the salt-free sterile Milli-Q H_2_O as the negative control, which was sequenced side by side with other samples, to analyze the kit for possible microbial contamination and to identify possible background contaminations of the salt samples. The results for the negative control showed that *Sulfitobacter* sp., *Enterococcus* sp., *Sediminicola* sp., *Bacillus* sp., and *Flavobacterium* sp. were the most frequent taxa. These bacteria have previously been reported as contaminants in DNA extraction kits and sequencing reagents (Glassing et al., 2016; Stinson et al., 2019). Other works have also found *Flavobacterium* sp. and *Bacillus* sp. in pure *Salmonella bongori* cultures to be linked to the contamination of the reagents (Salter et al., 2014). In the case of the genus *Sulfitobacter*, it seems likely that, when the control sample was processed alongside the salt samples, the sample material contaminated the negative control, similarly to what was previously observed (Edmonds and Williams, 2017); thus, this genus has a similar abundance with the Himalayan pink salt sample. In the case of the genus *Bacillus*, as this genus was not only detected by 16S rRNA gene metagenomics but was also cultured from all salt samples, this suggests that this genus is indeed present in table salts. It can be hypothesized that its presence in the negative control may be a result of a cross-contamination from the salt samples.

At the genus level, *Acinetobacter*, *Aquibacillus*, *Bacillus*, *Brevundimonas*, *Fictibacillus*, *Gracilibacillus*, *Halobacillus*, *Micrococcus*, *Oceanobacillus*, *Salibacterium*, *Salinibacter*, *Terribacillus*, and *Thalassobacillus* (bacteria) and *Haloarcula*, *Halobacterium*, and *Halorubrum* (archaea) were identified with both culture-dependent and culture-independent methods.

Microbiome sequencing revealed that those salts from marine origin shared a rather similar taxonomic profile. Archaeal taxa *Halorubrum*, *Halobacterium*, *Hallobellus*, *Natronomonas, Haloplanus*, *Halonotius*, *Halomarina*, and *Haloarcula* were prevalent in these three salts. The presence of *Halorubrum* sp., *Halobacterium* sp., *Haloarcula* sp., and *Halonotius* sp. were previously reported in the taxonomic profile of La Baleine Coarse sea salt, Palm Island black salt (United States), and food-grade salts from the Dead Sea (Israel) and Black Sea (Turkey) (Henriet et al., 2014). Moreover, a high diversity of archaeal genera *Halorubrum*, *Halobacterium*, *Haloarcula*, *Halonotius*, and *Natromonas* in two Korean marine salts was previously reported (Gibtan et al., 2017). The presence of other archaeal taxa, such as *Halarchaeum*, *Halomicrobium*, and *Salarchaeum*, was also detected in those Korean salts. This strongly suggests that the origin of salt plays a crucial role in establishing a similar microbial (mainly archaeal) population in table salts. Himalayan pink salt, Hawaiian black salt, and Viking salt showed more heterogeneous taxonomic profiles. Non-marine salts are usually enriched by additional minerals or flavors such as iron, hydrogen sulfate, activated charcoal, and dry vegetables. Previous research by Henriet et al. (2014) also confirmed that the archaeal diversity in Himalayan pink salt is lower compared to that of marine salts. Furthermore, genus *Sulfitobacter*, which belongs to the family *Rhodobacteraceae*, has previously been isolated from various habitats, like the Mediterranean Sea and hypersaline lakes, and organisms, such as starfish and seagrass (Prabagaran et al., 2007). *Sulfitobacter* sp. was the most abundant genus in Himalayan pink salt (57.5% of the microbial population). This genus was previously detected in various marine ecosystems and considered as sulfur-oxidizing chemoheterotrophic species (Yoon, 2019). Although 16S rRNA gene sequencing results showed *Sulfitobacter* sp. in Himalayan pink salt, no viable bacteria from this genus were isolated by culture-based methods. A possible reason for that may link to the lack of particular ions or minerals in the prepared media, which could affect the growth of some species.

Based on microbiome sequencing results, *Halorubrum* sp. is the most abundant archaea in the Atlantic salt, followed by *Halobellus*, *Halolamina*, and *Haloplanus*. All these genera (belonging to the class *Halobacteria*) are present in the other salts that originated from marine environments. *Halobacteria* is known to actively participate in processes of gene transfer and homologous recombination, and the population of the taxa in this class can increase very easily in saline and hyper-saline areas (Fullmer et al., 2014). As this taxonomic group is predominant in aquatic environments, it is not surprising that we detected these taxa within the Atlantic salt.

In the salt sample from Ibiza, the genus *Natronomonas* was the most frequent and abundant taxon, followed by *Haloquadratum*. The latter is frequent in solar salterns and is extremely halophilic. Only one species of this genus has been described so far, and it was isolated from Spain and Australia (Burns et al., 2007). Moreover, the previous research reported that *Haloquadratum* often grows in the company of some extremely halophilic bacteria such as *Salinibacter* (Antón et al., 2013), which we also identified together with *Haloquadratum* in samples from the supermarket and Ibiza salts. The genus *Salinibacter* has been isolated from saltern crystallizer ponds in Mediterranean locations such as Alicante and the Balearic Islands (Antón et al., 2002) as well as in other extremely salted areas in Argentina (Viver et al., 2018) and Iran (Makhdoumi-Kakhki et al., 2012). Interestingly, this bacterium shares some archaeal properties of the family *Halobacteriaceae* that thrives in the same habitat (Oren, 2013). In summary, *Halorubrum*, *Natronomonas, Haloquadratum*, and *Salinibacter* seem to be microbial markers for the salt samples obtained from marine environments.

The non-sea salt samples presented a taxonomic profile dominated by bacteria. The Himalayan salt was not isolated from the sea; it was extracted from the foothills of the Himalayan Mountain range that once were seas. The most important genus in this sample was *Sulfitobacter*. It might be hypothesized that *Sulfitobacter* appears in such a significant amount in this sample due to the activity that this genus shows to oxidize sulfites (Sorokin, 1995), which are present in high concentrations in the Himalaya region (Roy et al., 2020). Furthermore, this genus has previously been isolated from deep seawater (Song et al., 2019), where the salinity conditions are similar to those in the Himalayan.

In the black salt, the genus *Flavobacterium* stood out with 18.8% of abundance, a similar value to that detected in the supermarket marine salt. Other frequent halophilic bacteria were *Sulfitobacter*, *Yoonia-Loktanella*, or uncultured *Rhodobacteraceae.* The *Roseobacter* (*Yoonia-Loktanella*) group is a clade within the family *Rhodobacteraceae* that comprises a significant fraction of the total bacterial community on the ocean surface (Wirth and Whitman, 2018).

Another sample of the non-marine group is the Viking salt, which presents a high frequency for chloroplasts (more than 66% of its taxonomic profile) and mitochondria (4.7% of the total), which may be a consequence of its actual composition (it contains different vegetables). The genus *Bacillus* was also present in this sample. This genus contains certain salt-tolerant species (Sharma et al., 2015). Finally, both the Viking salt and black salt presented very similar frequencies for *Sulfitobacter* or *Yoonia-Loktanella*.

Table salt could theoretically be a carrier of human pathogens or health-promoting, probiotic strains, although the sensitivity of both to high NaCl concentrations has been previously reported (Ragul et al., 2017). In fact, by using 16S rRNA gene sequencing, some potentially pathogenic microbes such as *Enterococcus* sp. and *Brevundimonas* sp. were identified in almost all samples, whereas *Pseudomonas* sp. and *Lactobacillus* sp. were only detected in some table salts. No viable species from the genera *Enterococcus* and *Lactobacillus* was isolated by culture-based methods. However, several other potential pathogens from different taxa were recovered in this research. From those, *Brevundimonas diminuta* and *Bacillus circulans* were isolated from the Himalayan pink salt and supermarket salt, respectively. They were previously reported as the potent pathogens causing infection in immunocompromised and cancer patients (Han and Andrade, 2005; Alebouyeh et al., 2011).

Another interesting, yet poorly explored, connection between the microbial contents of the table salt and consumer health is the presence of halophilic and halotolerant taxa in the human gut, whose origin has not been determined to date, to the best of our knowledge. Indeed some halophilic and halotolerant microorganisms have previously been described by Seck et al. (2018) as “human halophilic microbiome.” At the genus level, it includes *Alkalibacterium* sp., *Bacillus* sp., *Chromohalobacter* sp., *Flavobacterium* sp., *Gracilibacillus* sp., *Halobacillus* sp., *Haloferax* sp., *Halomonas* sp., *Kocuria* sp., *Methanomassiliicoccus* sp., *Nesterenkonia* sp., *Oceanobacillus* sp., *Paenibacillus* sp., *Paucisalibacillus* sp., *Planococcus* sp., *Planomicrobium* sp., *Pseudomonas* sp., *Salinisphaera* sp., *Sciscionella* sp., *Shewanella* sp., *Sporosarcina* sp., *Terribacillus* sp., *Thalassobacillus* sp., *Vibrio* sp., and *Virgibacillus* sp. Another research by Oxley et al. (2010) reported that haloarchaea, besides methanogenic archaea, can be considered as members of the human microbiome, especially on the mucosa. Of all these taxa, we identified in at least one of the table salts analyzed the genera *Gracilibacillus* sp. (Himalayan pink salt), *Halobacillus* sp. (Atlantic salt, Himalayan pink salt, and Hawaiian black salt), *Oceanobacillus* sp. (Ibiza salt, Himalayan pink salt, and Viking salt), *Terribacillus* sp. (Viking salt), *Thalassobacillus* sp. (Atlantic salt, Ibiza salt, Himalayan pink salt, and supermarket salt), and *Virgibacillus* sp. (Himalayan pink salt and supermarket marine salt). Although the present work was not designed to confirm the colonization of the human gut by these taxa, it is clear that table salt can be a source of some of the halophilic bacteria that characterize either healthy or diseased human guts (Seck et al., 2018, 2019).

Some archaeal products such as bacterioruberin, bacteriorhodopsin, diether, and tetraether lipids are commercially available (Pfeifer et al., 2021). Moreover, the most common industrial enzymes (proteases, amylases, lipases, and esterase) are also produced by haloarchaea (Singh and Singh, 2017). Besides archaea, moderately and extremely halophilic bacteria can produce industrial hydrolytic enzymes. In this work, we have isolated some halophilic archaea and bacteria previously reported as excellent candidates for biotechnological applications. *Haloarcula hispanica* and *Haloarcula marismortui*, which both belonged to archaea (*Haloarcula* sp.), are considered as carotenoids and poly(3-hydroxybutyrate) (PHB) producers. PHB is commercially interesting as a primary substance for producing biodegradable plastics. Among the bacteria from our collection, *Bacillus megaterium*, isolated from Ibiza and Himalayan pink salt, is also considered a polyhydroxyalkanoate producer (Mohanrasu et al., 2020). Furthermore, *Bacillus flexus* was previously reported as a species that can degrade polyvinyl chloride, which is one of the most abundant petroleum-derived plastics on the earth (Giacomucci et al., 2019). *Brevibacterium frigoritolerans* was also isolated from Ibiza salt, previously reported as a bacterium with the potential to biodegrade organophosphorus insecticides such as phorate (Jariyal et al., 2015). The moderately halophilic bacterium *Thalassobacillus devorans* is also a promising candidate for phenol biodegradation (Garcia et al., 2005).

Taken together, our results show that table salts harbor a relatively diverse set of halophilic microorganisms. Salts of marine origin were dominated by archaeal lineages, whereas salts from salt mines or salt with added ingredients were dominated by bacteria. An important fraction of the identified microorganisms was culturable, and the biotechnological potential of some of them, as well as the potential pathogenicity of others, suggests that table salt is, beyond being a source of halophilic archaea, a relevant, poorly studied natural source of microbial diversity.

## Data Availability Statement

The datasets presented in this study can be found in online repositories. The names of the repository/repositories and accession number(s) can be found below: https://www.ncbi.nlm.nih.gov/bioproject/PRJNA701909.

## Author Contributions

LS, AG, and AL-P performed the experimental procedures and bioinformatic analysis, wrote, and approved the manuscript. MP designed and supervised the experimental work and wrote and approved the manuscript.

## Conflict of Interest

The authors declare that the research was conducted in the absence of any commercial or financial relationships that could be construed as a potential conflict of interest.

## Publisher’s Note

All claims expressed in this article are solely those of the authors and do not necessarily represent those of their affiliated organizations, or those of the publisher, the editors and the reviewers. Any product that may be evaluated in this article, or claim that may be made by its manufacturer, is not guaranteed or endorsed by the publisher.

## Footnotes

1
https://www.ezbiocloud.net
