# Supplementary material for: Beyond Archaea: The Table Salt Bacteriome
Source: Front Microbiol. 2021 Oct 29;12:714110. doi: 10.3389/fmicb.2021.714110 (PMC8586464; doi:10.3389/fmicb.2021.714110)
Supplement: Supplementary file 1 [file Data_Sheet_1.pdf]

Supplementary Material

Supplementary Table 1. Physical characters of table salt samples and ingredients. All salts lacked added iodine. Atlantic salt, Ibiza salt, and Supermarket salt originated from marine environments. Atlantic salt has high natural minerals and trace elements. Ibiza salt contains more than 80 essential minerals and trace elements, such as magnesium, selenium and fluorine. Himalaya pink salt extracted from the foothill of Himalaya alpine, where the layers of sediments from an ancient ocean formed during the past 200 million years. Himalayan black salt is unrefined mineral rock salt extracted from the Himalayan Mountain range with less NaCl Content. They are rich in iron and hydrogen sulfide, respectively. Hawaiian black salt contains up to 2 % activated carbonate. Viking salt is supplemented with smoked flavor, black pepper, turmeric, dextrose and dried onion**.**

Name of Salt	Color	Particle Size (mm)	Origin or packaging	Total salt Content (%)	Sodium content	Package Size (g)
**Atlantic salt**	White	1.0-2.0	Spain	-	-	125
**Ibiza salt**	White	0.5-1.0	Spain	90.5	-	150
**Himalaya pink salt**	Pink	1.0-2.0	Pakistan	-	-	250
**Black salt**	Black	1.5-4.0	Hawaii	98.97	39.59 g	100
**Viking salt**	Yellow	0.1-0.5	France	-	25.49 mg	100
**Himalaya black salt**	Pinkish Gray	1.0-5-0	Pakistan	-	-	180
**Supermarket salt**	White	0.5-3.0	Spain	< 90	-	125

Supplementary table 2. Identification of bacteria and archaea from different table salts based on genus and species level, and source of isolation.

Genus	Species level	Source of Isolation
Acinetobacter	*A. vivianii*	Ibiza salt
Alkalihalobacillus	*A. berkeleyi*	Supermarket salt
	A. hwajinpoensis	Ibiza salt and Supermarket salt
Aquibacillus	*A. koreensis*	Supermarket salt
Bacillus	*ABCF_s*	Atlantic salt, Himalayan pink salt and supermarket salt
	B. aequororis	Atlantic salt
	B. aidingensis	Himalayan pink salt
	B. altitudinis	Viking salt
	B. aryabhattai	Himalayan pink salt
	B. cabrialesii	Viking salt
	B. circulans	Himalayan pink salt
	B. endophyticus	Ibiza salt
	B. filamentosus	Ibiza salt and Viking salt
	B. flexus	Ibiza salt
	B. infantis	Atlantic salt
	B. licheniformis	Himalayan pink salt and Viking salt
	B. luteolus	Black salt
	B. megaterium	Ibiza salt and Himalayan pink salt
	B. onubensis	Ibiza salt
	B. oryzaecorticis	Atlantic salt
	B. paralicheniformis	Himalayan pink salt and Viking salt
	B. pumilus	Viking salt
	B. salidurans	Supermarket salt
	B. sonorensis	Himalayan pink salt
	B. subtilis	Viking salt
	B. tequilensis	Atlantic salt, Himalayan pink salt and Viking salt
	B. thaonhiensis	Himalayan pink salt
	B. timonensis	Viking salt
	B. velezensis	Viking salt
	B. zanthoxyli	Atlantic salt and Himalayan pink salt
	B. zhangzhouensis	Himalayan pink salt and Viking salt
	Brevibacterium frigoritolerans	Ibiza salt
Brevundimonas	*B. diminuta*	Supermarket salt
Cytobacillus	*C. firmus*	Atlantic salt
	C. oceanisediminis	Ibiza salt and Himalayan pink salt
Dermacoccus	*D. profundi*	Himalayan pink salt
Fictibacillus	*F. nanhaiensis*	Atlantic salt
Gracilibacillus	*G. dipsosauri*	Himalayan pink salt
	G. salitolerans	Himalayan pink salt
**EU817569_g**	EU817569_s	Supermarket salt
Haloarcula	*H. hispanica*	Atlantic salt
	H. marismortui	Atlantic salt
Halobacillus	*H. alkaliphilus*	Atlantic salt
	H. dabanensis	Himalayan pink salt
	H. hunanensis	Himalayan pink salt
	H. litoralis	Atlantic salt and Himalayan pink salt
	H. sediminis	Black salt
	H. trueperi	Atlantic salt
Halobacterium	*H. hubeiense*	Atlantic salt and Himalayan pink salt
Halorubrum	*H. salinum*	Ibiza salt
	H. sodomense	Ibiza salt
	H. xinjiangense	Ibiza salt

Lentibacillus	*L. jeotgali*<br>*L. juripiscarius*<br>*L. lacisalsi*<br>*L. salarius*<br>*L. salicampi*	Himalayan pink salt<br>Ibiza salt<br>Himalayan pink salt<br>Himalayan pink salt<br>Ibiza salt and Himalayan pink salt
Mesobacillus	*M. subterraneus*	Atlantic salt
Metabacillus	*M. endolithicus*<br>*M. halosaccharovorans*	Ibiza salt<br>Atlantic salt, Ibiza salt, and Supermarket salt
Micrococcus	*M. luteus*	Atlantic salt
Oceanobacillus	*O. kimchii*<br>*O. picturae*	Ibiza salt and Viking salt<br>Himalayan pink salt
Peribacillus	*P. simplex*	Ibiza salt
Piscibacillus	*P. halophilus*<br>*P. salipiscarius*	Himalayan pink salt<br>Himalayan pink salt
Pontibacillus	*P. salipaludis*<br>*P. yanchengensis*	Atlantic salt<br>Atlantic salt
Salibacterium	*S. halotolerans*<br>*S. nitratireducens*	Himalayan pink salt<br>Atlantic salt
Salinibacter	*S. ruber*	Ibiza salt
**Staphylococcus**	*S. epidermidis*	Black salt
Terribacillus	*T. halophilus*	Viking salt
Thalassobacillus	*T. cyri*<br>*T. devorans*<br>*T. hwangdonensis*	Atlantic salt, Ibiza salt, Himalayan pink salt, and supermarket salt<br>Ibiza salt, Himalayan pink salt, and supermarket salt<br>Atlantic salt and Himalayan pink salt
Virgibacillus	*V. byunsanensis*<br>*V. dakarensis*<br>*V. kapii*	Supermarket salt<br>Himalayan pink salt<br>Himalayan pink salt

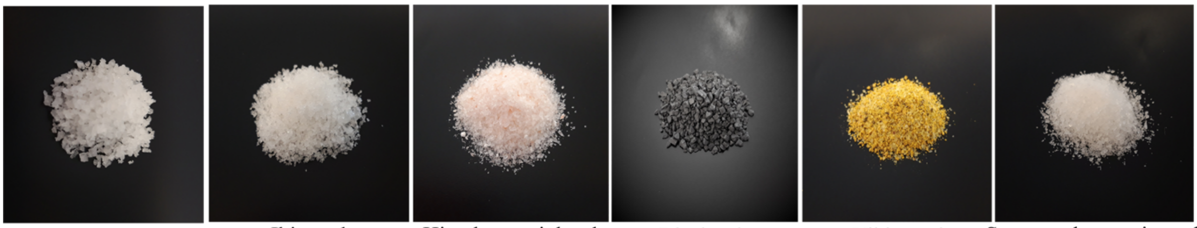

Atlantic salt | Ibiza salt | Himalayan pink salt | Black salt | Viking salt | Supermarket marine salt

Supplementary Figure 1. Six table salts samples from different manufacturers are analyzed in this study.

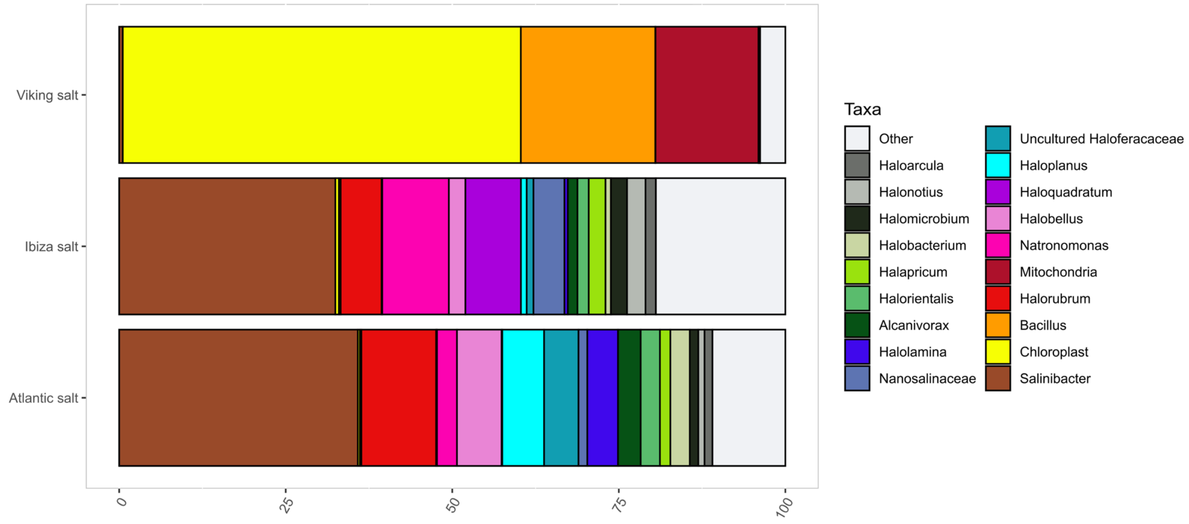

Supplementary Figure 2. Relative abundance of the microbial diversity from Atlantic, Ibiza, and Viking salt by using bacterial designed primers. These three table salts were richer in bacteria in comparison with the previous NGS analysis.
